# Standardizing Upper Abdominal Fascial Terminology: A Transdisciplinary Expert Consensus From the Japanese Society for Endoscopic Surgery

**DOI:** 10.1111/ases.70348

**Published:** 2026-07-09

**Authors:** Jun Watanabe, Hisashi Shinohara, Kazutaka Obama, Takahiro Kinoshita, Yuichi Nagakawa, Kohei Nakata, Tsunenori Kondo, Jun Miki, Hiroyuki Egi, Yoshiharu Sakai, Yuko Kitagawa

**Affiliations:** ^1^ Department of Colorectal Surgery Kansai Medical University Hirakata Japan; ^2^ Department of Gastroenterological Surgery Hyogo Medical University Nishinomiya Hyogo Japan; ^3^ Department of Surgery, Graduate School of Medicine Kyoto University Kyoto Japan; ^4^ Gastric Surgery Division National Cancer Center Hospital East Kashiwa Japan; ^5^ Department of Gastrointestinal and Pediatric Surgery Tokyo Medical University Tokyo Japan; ^6^ Department of Surgery and Oncology, Graduate School of Medical Sciences Kyushu University Fukuoka Japan; ^7^ Department of Urology, Adachi Medical Center Tokyo Women's Medical University Tokyo Japan; ^8^ Department of Urology, Kashiwa Hospital The Jikei University School of Medicine Chiba Japan; ^9^ Department of Surgery Kitasato University Medical Center Saitama Japan; ^10^ Cancer Center, Uji Tokusyukai Medical Center Kyoto Japan; ^11^ Department of Surgery Keio University School of Medicine Tokyo Japan

## Abstract

**Background:**

Terminology describing fascial structures in the upper abdomen remains inconsistent across surgical specialties. Eponymous structures such as the fasciae of Toldt, Gerota, Fredet, and Treitz are widely used, but their definitions vary among disciplines. To address this issue, the Japanese Society for Endoscopic Surgery established a transdisciplinary surgical working group to standardize intraoperative fascial nomenclature in the upper abdomen.

**Methods:**

A transdisciplinary surgical expert panel comprising surgeons from upper gastrointestinal, colorectal, hepatopancreatobiliary, and urological specialties held structured consensus meetings between February and September 2023. Surgeons from each field presented intraoperative anatomical structures with inconsistent terminology. Through iterative discussions based on intraoperative findings and surgical anatomy, consensus statements were developed.

**Results:**

Four consensus statements were established. First, the renal fascia (Gerota fascia) was recognized as a representative relatively dense condensation fascia that can be consistently identified intraoperatively. Second, structures traditionally referred to as the fasciae of Toldt, Fredet, and Treitz were interpreted as regional or topographic expressions of fusion fascia, rather than independent anatomical membranes. Fusion fascia was understood as a loose, multilayered fusion‐related connective tissue zone within the broad operative anatomical spectrum of fascia. Third, because fusion fascia is multilayered, defining a single fixed surgical interface was considered inappropriate. Fourth, membrane‐like structures exposed on the visceral side after dissection of the fusion fascia were collectively designated as proper visceral fasciae.

**Conclusions:**

This surgeon‐led consensus provides an operative nomenclature framework for fascial structures recognized intraoperatively in upper abdominal endoscopic surgery. The three core terms may serve as a basic conceptual skeleton for describing upper abdominal fascial anatomy and facilitate cross‐specialty communication.

## 1 Introduction

The anatomical relationship between the mesentery and the retroperitoneum has long been debated. In 1879, Toldt described persistence of the colonic mesentery and its interface with the retroperitoneum, referring to the boundary layer as the *lamina mesenterii propria* [1]. In contrast, Treves later proposed the concept of retroperitoneal fixation [2], which became the dominant paradigm and influenced surgical understanding for decades. In 1895, Gerota described the anterior renal fascia, providing a clearer framework for retroperitoneal anatomy [3]. Despite this, the mesentery–retroperitoneum relationship continued to be interpreted in terms of peritoneal fusion. In 1984, Goligher revisited Toldt's observations and introduced the concept of the fascia of Toldt as a fusion fascia between the mesocolon and retroperitoneum [4], renewing interest in the fascial planes encountered during colonic mobilization.

The modern mesentery‐based surgical paradigm was established with the introduction of total mesorectal excision and complete mesocolic excision [5, 6]. These techniques emphasize sharp dissection along embryologically defined planes, recognizing the mesentery as a distinct anatomical and oncological unit [7], and have improved oncological outcomes in colorectal cancer surgery.

Terminology describing fascial structures in the upper abdomen remains heterogeneous. Terms such as the fascia of Toldt, Treitz, and Fredet, as well as posterior pancreatic fascia, mesenteric fascia, and mesocolic fascia, are used variably across surgical disciplines. In addition, multiple conceptual models—including mesofascial, colofascial, and retrofascial interfaces—have been proposed [8, 9]. These terms are often applied inconsistently and may reflect differences in surgical perspective rather than distinct anatomical entities, leading to variability in anatomical description and operative communication [8, 9].

Recent anatomical and developmental studies have challenged the classical concept of fusion fascia as a discrete membranous structure. While earlier models proposed mesothelial fusion or retention with intervening connective tissue [10, 11, 12, 13], newer evidence suggests that mesothelial displacement during mesenteric adhesion, rather than true peritoneal fusion, forms the mesenteric–retroperitoneal interface [14]. This mesothelial retreat theory further proposes that this interface develops as a continuous extraperitoneal connective tissue layer, with Toldt's fascia representing part of this continuum [15]. These findings support reconsidering fusion fascia as a continuous connective tissue interface rather than a distinct membrane, indicating that traditional interpretations of fascial anatomy require revision.

To address these issues, the Japanese Society for Endoscopic Surgery (JSES) established a multidisciplinary surgical working group comprising experts from upper gastrointestinal, colorectal, hepatopancreatobiliary, and urological surgery. The working group sought to clarify the intraoperative nomenclature and conceptual framework of upper abdominal fascial anatomy across surgical subspecialties.

## 2 Methods

### 2.1 Study Design and Working Group Formation

This study was designed as a surgeon‐led consensus on intraoperative anatomical terminology. The working group was multidisciplinary across surgical subspecialties, comprising experts from upper gastrointestinal, colorectal, hepatopancreatobiliary, and urological surgery with two representatives selected from each field. The group focused on terminology for fascial structures and related connective tissue planes recognized intraoperatively, particularly in endoscopic surgery. The working group held eight structured meetings organized by JSES between February and September 2023. Panel members proposed anatomical structures with inconsistent or unclear terminology or conceptual interpretation across specialties, and those most relevant to surgical practice and interdisciplinary communication were selected for further discussion. As this work is a consensus study without patient data, formal ethical approval and informed consent were not required.

### 2.2 Consensus Development Process

For each anatomical structure, experts from relevant specialties presented their interpretations, followed by structured multidisciplinary discussions to compare terminology and concepts. Through iterative revisions, draft statements were refined until general agreement was achieved. Consensus was defined as agreement among working group members after discussion and revision.

This consensus was primarily intended to establish shared nomenclature for surgical anatomy recognized intraoperatively, rather than to provide a strict histological definition of fascia. In this manuscript, the term fascia is used in a broad operative anatomical sense, encompassing a spectrum of connective tissue structures encountered during surgical dissection. Embryological, histological, and anatomical findings from previous studies were used as cited background to support the operative terminology, but no new embryological or histological validation was performed in this study.

### 2.3 Formulation of Consensus Statements

Based on the discussions within the expert panel, the working group formulated consensus statements addressing the nomenclature and conceptual interpretation of fascial structures encountered in upper abdominal surgery.

These statements focused on four key aspects of surgical anatomy:

1. the nomenclature and recognition of fascia, with particular emphasis on the definition of the renal fascia:
2. the conceptual interpretation of fusion fascia;
3. the interpretation of surgical dissection interfaces; and
4. the nomenclature of visceral membrane‐like structures exposed after dissection of the fusion fascia.

## 3 Results

Following iterative discussion and revision, the expert panel reached agreement on four key consensus statements regarding the nomenclature and recognition of fascial structures in upper abdominal surgery (Table 1).

**TABLE 1:** Summary of the consensus statements on fascial nomenclature in the upper abdomen.

Consensus statement	Topic	Consensus summary
1	Nomenclature and recognition of fascia	The term fascia is used in a broad operative anatomical sense, encompassing a spectrum of connective tissue structures encountered during surgical dissection. Within this spectrum, renal fascia (Gerota fascia) was identified as a representative relatively dense condensation fascia that can be consistently recognized intraoperatively as a thick membrane‐like structure. The panel also agreed that the subperitoneal fascia, lateroconal fascia, and renal fascia form a continuous retroperitoneal fascial system.
2	Conceptual interpretation of fusion fascia	Fusion fascia should not be interpreted as a discrete membranous structure, but as a loose, multilayered connective tissue zone formed through developmental adhesion between mesenteric structures and the retroperitoneum within the broad operative anatomical spectrum of fascia. Structures traditionally referred to as the fasciae of Toldt, Treitz, and Fredet are not independent anatomical fasciae, but can be interpreted as regional or topographic expressions of fusion fascia.
3	Interpretation of surgical dissection interfaces	Because fusion fascia consists of a loose, multilayered connective tissue zone, a single anatomically fixed interface does not exist. Instead, several potential dissection planes may be present within this zone. An interface should therefore be understood as a surgically selected dissection plane within the fusion fascia rather than as a distinct anatomical structure.
4	Nomenclature of visceral membrane‐like structures exposed after dissection of the fusion fascia	Membrane‐like structures exposed on the visceral side after dissection within fusion fascia do not necessarily represent pre‐existing independent anatomical fasciae, but rather surgically revealed or accentuated visceral‐side surfaces. The working group adopted the term *proper visceral fasciae* as an operative collective designation for these surgically recognized membrane‐like structures including the mesocolic fascia and fascial surfaces traditionally referred to as the anterior and posterior pancreatic fascia.

*Note:* The principal aim of this consensus is to establish shared nomenclature for surgical anatomy recognized intraoperatively, particularly in endoscopic surgery, rather than to provide a strict histological definition of fascia.

### 3.1 Consensus Statement 1—Nomenclature and Recognition of Fascia

The working group agreed that the term fascia should be used in a broad operative anatomical sense, encompassing a spectrum of connective tissue structures encountered during surgical dissection, rather than being restricted to clearly identifiable dense membrane‐like structures. Within this spectrum, the renal fascia (Gerota fascia) was considered a representative relatively dense condensation fascia in the upper abdominal retroperitoneum. Because of its relatively dense connective tissue nature, the renal fascia can be consistently recognized intraoperatively as a thick membrane‐like structure. Originally described by Gerota in 1895, the renal fascia forms a well‐defined connective tissue layer surrounding the perirenal space and can be consistently identified during surgical dissection.

The panel further agreed that the subperitoneal fascia, lateroconal fascia, and renal fascia represent a continuous fascial system within the retroperitoneum. In surgical practice, the structure often referred to as the “retroperitoneal fascia (後腹膜下筋膜)” by colorectal surgeons corresponds anatomically to the renal fascia. Accordingly, the term *renal fascia* was considered preferable when referring to this structure in surgical anatomy.

### 3.2 Consensus Statement 2—Conceptual Interpretation of Fusion Fascia

The working group discussed the concept of fusion fascia, including structures traditionally referred to as the fascia of Toldt, Treitz, and Fredet. Based on anatomical and surgical observations, the panel agreed that fusion fascia should not be interpreted as a discrete membranous structure. Rather, within the broad operative anatomical spectrum of fascia, it should be understood as a loose, multilayered fusion‐related connective tissue zone formed between mesenteric structures and the retroperitoneal space. The description of fusion fascia as multilayered refers to its morphological interpretation based on previous anatomical and histological studies. From an operative perspective, this multilayered connective tissue zone may contain several potential dissection planes, allowing surgeons to select an appropriate plane according to surgical strategy and local anatomy.

Accordingly, the structures traditionally referred to as the fascia of Toldt, Treitz, and Fredet should not be regarded as separate anatomical fasciae, but rather should all be interpreted as forms of fusion fascia.

### 3.3 Consensus Statement 3—Interpretation of Surgical Dissection Interfaces

The working group further examined the concept of the surgical interface between fascial structures. Because fusion fascia consists of a loose, multilayered connective tissue, a single anatomically fixed interface does not exist. Instead, several potential dissection planes may be present within this zone. From this perspective, the term *interface* should not be interpreted as a distinct anatomical structure. Rather, it should be understood as a surgically selected dissection plane within the fusion fascia. The panel therefore agreed that assigning a unique anatomical name to a single interface within fusion fascia is not appropriate.

### 3.4 Consensus Statement 4—Nomenclature of Visceral Membrane‐Like Structures Exposed After Dissection of the Fusion Fascia

The working group discussed the nomenclature of membrane‐like structures that become apparent on the visceral side after dissection of the fusion fascia. These structures were considered not necessarily to represent pre‐existing independent anatomical fasciae, but rather surgically revealed or accentuated visceral‐side membrane‐like surfaces that become apparent after separation of loose connective tissue layers within the fusion fascia. The working group acknowledged that these surfaces may include a portion of the dissected fusion fascia remaining on the visceral side. From an intraoperative perspective, their recognition was considered clinically important because both the loose connective tissue encountered during dissection and the membrane‐like structure appearing on the visceral side after completion of dissection may serve as landmarks indicating that dissection has proceeded along an oncologically appropriate plane.

Following discussion, the panel agreed to adopt the term *proper visceral fasciae* as a collective designation for these membrane‐like structures exposed on the visceral side after dissection of the fusion fascia. This term was defined as encompassing fascial surfaces associated with individual visceral organs, including the mesocolic fascia and related organ‐specific fascial surfaces. The Japanese equivalent zōsoku koyū kinmaku (臓側固有筋膜) was proposed as the collective designation corresponding to *proper visceral fasciae*. Here, “proper” is used in an operative sense, referring to the visceral or mesenteric side of the surgically defined resection package, and not to a pre‐existing organ‐intrinsic fascia. The intended parallel with the fascia propria of the rectum is therefore operative, based on its position on the visceral side of the surgical envelope, rather than developmental or histological. With this terminology, the membrane‐like structure that becomes apparent when the right or left mesocolon is dissected free from the retroperitoneum, with part of the fusion fascia remaining attached, can be described as the proper visceral fascia. Similarly, fascial structures that have traditionally been referred to as the posterior pancreatic fascia or anterior pancreatic fascia may also be interpreted as membrane‐like structures exposed by dissection of the fusion fascia and can therefore be described as forms of proper visceral fascia.

**FIGURE 1:**
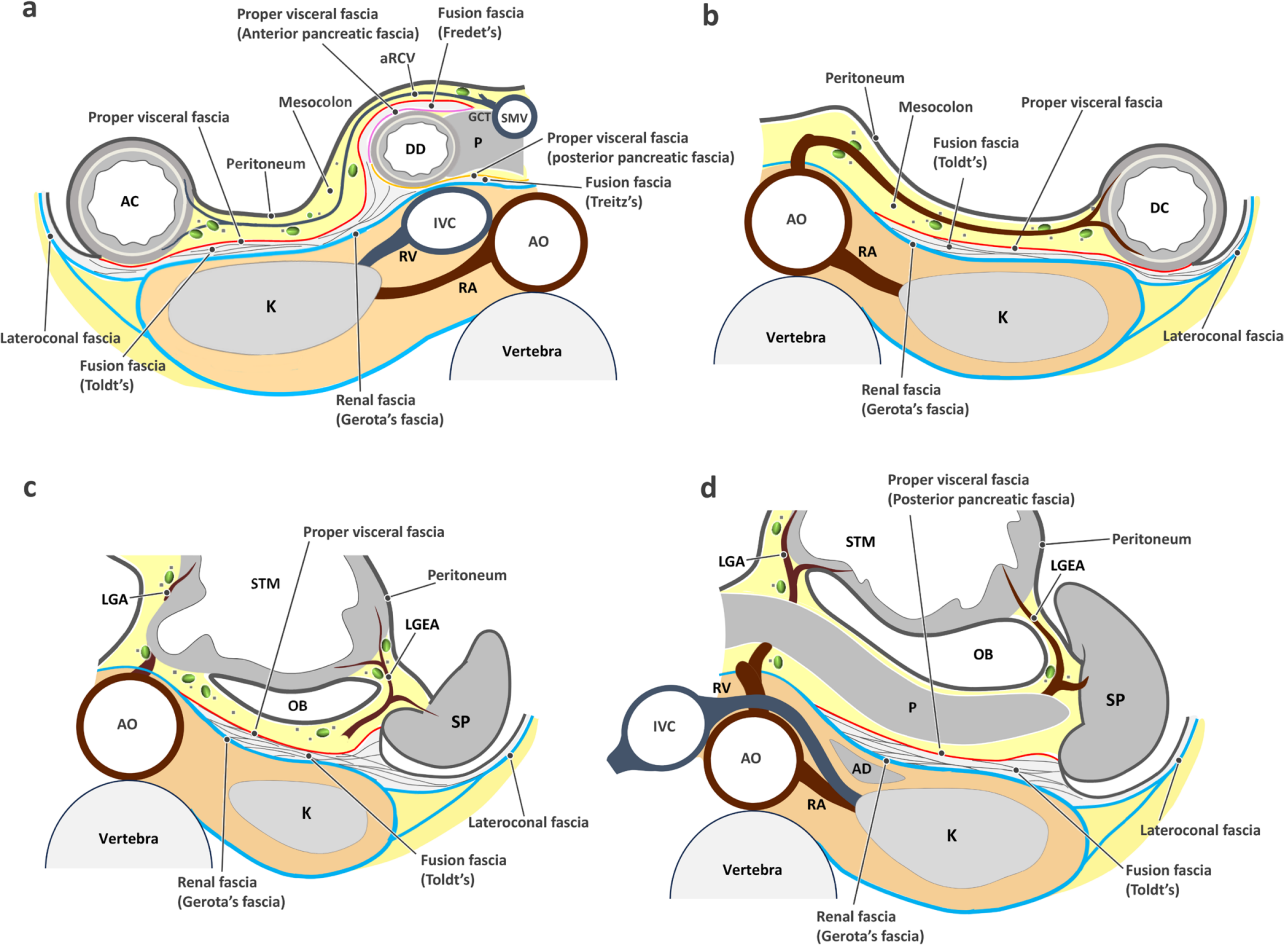
Updated schematic representation of the fusion interface between visceral organs and the retroperitoneum. Traditional eponymous or organ‐specific terms shown in parentheses are provided only as supplementary descriptors to help readers familiar with conventional terminology identify the corresponding anatomical regions, and do not indicate independent anatomical fascia. (a) The fusion interface among the ascending mesocolon, the pancreaticoduodenal region (pancreatic head and duodenum), and the retroperitoneum. (b) The fusion interface between the descending mesocolon and the retroperitoneum. (c) The fusion interface between the mesogastrium (including the spleen) and the retroperitoneum. (d) The fusion interface between the mesogastrium (including the distal pancreas and spleen) and the retroperitoneum. The renal fascia and its continuous extension, the lateroconal fascia, are depicted in light blue. The fusion fascia is illustrated not as a single discrete membrane, but rather as a multilayered connective tissue zone that permits the selection of surgical dissection planes. The *proper visceral fascia*, newly proposed as a collective designation for membrane‐like structures exposed on the visceral side following dissection of the fusion fascia, is depicted in red. AC, ascending colon; AD, adrenal gland; AO, aorta; aRCV, accessory right colic vein; DC, descending colon; DD, duodenum; GCT, gastrocolic trunk; IVC, inferior vena cava; K, kidney; LGA, left gastric artery; LGEA, left gastroepiploic artery; OB, omental bursa; P, pancreas; RA, renal artery; RV, renal vein; SMV, superior mesenteric vein; SP, spleen; STM, stomach.

**FIGURE 2:**
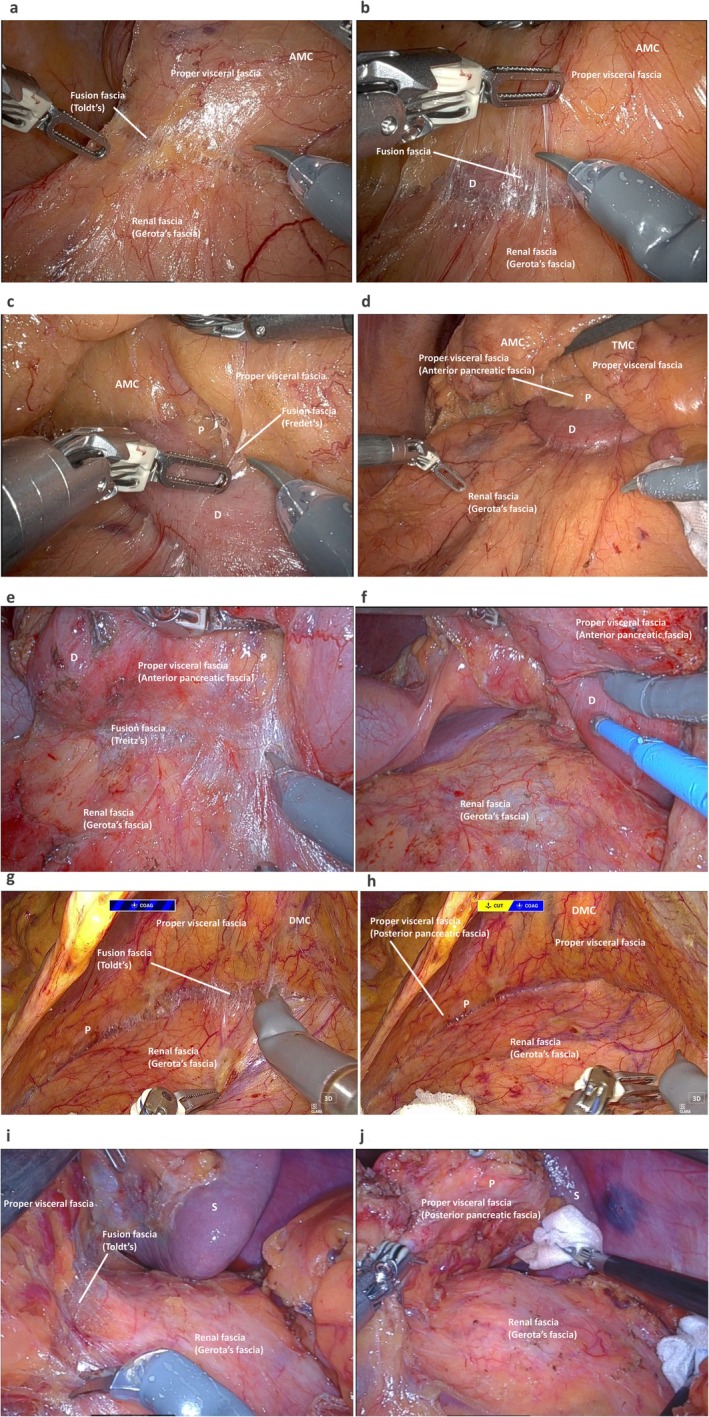
Intraoperative photographs demonstrating the dissection of the right‐sided mesocolon from the right retroperitoneum and the pancreaticoduodenal region (a–d); dissection of the mesoduodenum, including the pancreatic head, from the right retroperitoneum (e, f); dissection of the left‐sided mesocolon and distal pancreas from the left retroperitoneum (g, h); and dissection of the mesogastrium (MG), including the distal pancreas, from the left retroperitoneum (i, j). AMC, ascending mesocolon; D, duodenum; DMC, descending mesocolon; P, pancreas; SP, spleen; TMC, transverse mesocolon.

Figure 1 presents a schematic of the fusion interface between visceral organs and the retroperitoneum based on this consensus, using established anatomical terminology except for the newly proposed term *proper visceral fascia*. Figure 2 illustrates the application of this updated nomenclature in intraoperative settings.

## 4 Discussion

This consensus initiative addressed the lack of standardized terminology for fascial structures in upper abdominal surgery. Although surgeons across specialties dissect along similar planes, different terms are often used to describe the same structures, reflecting not only semantic variation but also underlying differences in anatomical interpretation.

The primary contribution of this consensus is the organization of intraoperative anatomical terminology for upper abdominal fascial structures across surgical subspecialties. The embryological and histological concepts discussed in this manuscript, including the mesothelial retreat theory and previous descriptions of renal fascia and fusion fascia, should therefore be understood as cited background supporting the terminology, rather than as independent anatomical or developmental conclusions generated by the present working group. Moreover, to avoid conceptual ambiguity, the present consensus explicitly uses the term fascia as a broad operative anatomical spectrum rather than as a single uniform histological category. Within this spectrum, renal fascia, fusion fascia, and proper visceral fasciae represent different structural and operative concepts. The renal fascia represents a relatively dense condensation fascia that can be consistently recognized intraoperatively as a thick membrane‐like structure. Fusion fascia refers to the broader loose, multilayered fusion‐related connective tissue zone between visceral mesenteries and the retroperitoneal space, through which surgical dissection is performed. Proper visceral fasciae refer to the visceral‐side membrane‐like surface or envelope that becomes apparent after dissection within this zone. Thus, these three terms should not be understood as identical anatomical entities, but as practical operative anatomical terms describing different aspects of the fascial spectrum encountered during endoscopic surgery.

The renal fascia consists of anterior and posterior components, originally described by Gerota and Zuckerkandl, and later came to be collectively known as “Gerota's fascia” [3, 16, 17]. In Gerota's original publication, this fascia was depicted as a distinct membranous structure, emphasizing that it was already macroscopically recognizable with the anatomical techniques available at that time. The panel further agreed that the subperitoneal fascia, lateroconal fascia, and renal fascia represent a continuous fascial system within the retroperitoneum [18, 19]. Some earlier interpretations regarded this fascia as a surgical artifact generated by traction and condensation of loose connective tissue during dissection [20]. However, increasing anatomical and radiological evidence suggests otherwise. Fetal studies identified the renal fascia as a dense fibrous structure distinct from the mesocolon before surgical manipulation, and cadaveric CT studies demonstrated it as a consistent high‐density line [21, 22]. Developmental observations, including the reported absence or alteration of renal fascia in renal agenesis and its persistence in pelvic kidneys, suggest that renal fascia formation is closely linked to renal growth and migration during embryogenesis [23]. Accordingly, Gerota's fascia should be regarded not as a surgically generated membrane, but as a representative relatively dense condensation fascia that can be consistently identified intraoperatively. Importantly, this interpretation does not imply that Gerota's fascia represents the only legitimate or “true” form of fascia in the abdomen; rather, it should be understood as one well‐defined example within the broad operative anatomical spectrum of fascia. This interpretation is also strongly supported by the stable and magnified visualization provided by modern robotic surgery [11].

This distinction has important implications for surgical interpretation. Historically, structures such as the fascia of Toldt, Fredet, and Treitz have often been described as separate fascial membranes [9, 24]. However, fusion fascia is more appropriately understood not as a discrete membranous structure, but as a multilayered loose connective tissue zone formed through developmental adhesion between mesenteric structures and the retroperitoneum [11, 25, 26]. On this basis, the present working group considered that these eponymous structures most likely represent different regional expressions of the same developmental adhesion process occurring between mesenteric and retroperitoneal tissues. This interpretation is in line with recent proposals that, whenever possible, eponym‐based terminology should be replaced by more topographically and anatomically precise terms [9, 11, 27]. From this perspective, the terms Toldt, Fredet, and Treitz may remain useful as locational descriptors, but they do not denote fundamentally distinct fascial entities and can all be interpreted as forms of fusion fascia.

Another important point of discussion was the concept of the surgical dissection interface. The concept of a single predefined dissection “interface” between the mesocolon and retroperitoneum has been widely described [8, 9]. However, the panel emphasized that fusion fascia is a multilayered structure, allowing multiple potential dissection planes. Accordingly, the retrocolic fascial system should be viewed as a layered region rather than a fixed boundary [11], and dissection occurs within this structure depending on surgical strategy and local anatomy.

The working group also discussed the ambiguity surrounding organ‐specific fascial structures that become apparent when loose connective tissue from the fusion fascia remains attached after dissection—structures that have variably been termed mesocolic fascia, fascia propria, and other related names, but for which no unified terminology exists [9, 15]. In colorectal surgery, the importance of preserving the mesocolic envelope during CME has been repeatedly emphasized from both technical and oncological perspectives [6, 28, 29]. Histological studies have further shown that preservation of the mesocolic plane correlates with specimen quality and may be associated with improved oncological outcomes [28, 30]. At the same time, however, the precise structural identity of the membrane‐like layer covering the mesocolon remains debated. Some authors have described it as mesocolic fascia or mesenteric fascia [9, 31, 32], whereas others have suggested that this membrane becomes apparent only after dissection and may reflect a surgically accentuated layer rather than a pre‐existing anatomical fascia [11, 12, 15, 33]. The present consensus supports the latter interpretation, while also acknowledging the practical value of recognizing this layer during surgery.

This issue led to extensive discussion regarding the nomenclature of membrane‐like structures exposed on the visceral side after dissection of the fusion fascia. In the early phase of the consensus discussions, the working group considered whether the term *visceral fascia* might serve as an appropriate general designation for these structures. However, this term has already been used broadly in anatomical literature and in *Terminologia Anatomica* to refer to a wide spectrum of connective tissue structures associated with visceral organs, including elements of the subperitoneal fascia [34, 35]. Its unqualified use in a narrower surgical context would therefore risk creating further ambiguity. To address this problem, the working group adopted the term *proper visceral fasciae* as a collective designation for membrane‐like structures that become apparent on the visceral side after dissection of the fusion fascia. This terminology intentionally parallels the established term *fascia propria of the rectum* [36, 37], while extending the concept to other organs, including the mesocolon and pancreas. With this terminology, not only the membrane‐like structure that becomes apparent when the right or left mesocolon is detached from the retroperitoneum, but also structures traditionally referred to as the posterior pancreatic fascia or anterior pancreatic fascia [9], can be interpreted as membrane‐like structures exposed after dissection of the fusion fascia and therefore collectively described as proper visceral fasciae. To avoid conceptual confusion, fusion fascia and proper visceral fasciae were distinguished according to their operative meanings within the broad fascial spectrum. The working group acknowledged that the proper visceral fasciae may include a portion of the dissected fusion fascia remaining on the visceral side. However, from an intraoperative perspective, this distinction is useful because both the loose connective tissue encountered during dissection and the visceral‐side membrane‐like surface appearing after completion of dissection may serve as important landmarks indicating preservation of the visceral or mesenteric envelope and dissection along an oncologically appropriate plane, particularly in endoscopic surgery. Therefore, the clinical relevance of proper visceral fasciae should be understood as an operative landmark of surgical plane recognition, rather than as evidence of an independent histological entity.

The present consensus should be understood as complementary to the recent review by Shinohara et al., which provided the anatomical and conceptual framework for fascial anatomy, including the mesothelial retreat theory, renal fascia as a condensation fascia, and the practical use of “fusion fascia” as a descriptive surgical term [11]. The present consensus translates this framework into a JSES surgeon‐led terminology consensus for intraoperatively recognized anatomy across surgical subspecialties. A concrete example is the introduction of “proper visceral fasciae” as a standardized umbrella term for the membranous coverings exposed on the deep surface of the mesentery after dissection of the fusion fascia. This directly addresses the terminological gap noted by Shinohara et al., who provisionally referred to this structure as “fascia propria” following Chen et al. [15]. Thus, the two papers should be regarded as sequentially complementary: Shinohara et al. established the anatomical framework, whereas the present consensus formalizes the operative terminology required for its practical application in surgery.

This study has several limitations. The conclusions are based on expert consensus without systematic review or histological validation, and the process, conducted within the Japanese surgical community, may not fully reflect international terminology. Nevertheless, the multidisciplinary panel provides a valuable transdisciplinary perspective.

In conclusion, this transdisciplinary surgical consensus provides an operative nomenclature framework for fascial structures recognized intraoperatively in upper abdominal endoscopic surgery. Rather than providing a strict histological definition of fascia or new embryological validation, this framework uses fascia as a broad operative anatomical spectrum and organizes renal fascia, fusion fascia, and proper visceral fasciae as practical operative anatomical terms representing different structural and operative concepts. Adoption of the three core terms may provide a basic conceptual skeleton for describing upper abdominal fascial anatomy. In detailed intraoperative descriptions, additional anatomical context related to the organ, region, or procedure may be necessary for more precise specification. This framework may facilitate communication across surgical subspecialties and promote a more consistent understanding of surgical anatomy in minimally invasive surgery.

## Author Contributions

J.W. conceived the study, organized and coordinated the working group, led the consensus process, and prepared the first draft of the manuscript. Y.S., Y.K. supervised the study. H.S., K.O., T.K., Y.N., K.N., T.Ko., J.M., H.E., and Y.S. contributed to the multidisciplinary expert discussions and to the formulation of the consensus statements. All authors participated in the consensus meetings, contributed to the interpretation of the findings, reviewed the manuscript, and approved the final version.

## Funding

The authors have nothing to report.

## Conflicts of Interest

J.W. reported receiving honoraria for lectures from Covidien Japan, Johnson and Johnson, Eli Lilly, Terumo Corporation, and Takeda Pharmaceuticals, and receiving research funding from Covidien Japan, Stryker Japan, and Terumo Corporation outside of the submitted work. The other authors declare no conflicts of interest.

## Data Availability

Data sharing not applicable to this article as no datasets were generated or analyzed during the current study.
